# Extended-Spectrum β-Lactamases (ESBL) Producing Bacteria in Animals

**DOI:** 10.3390/antibiotics12040661

**Published:** 2023-03-28

**Authors:** Chien-Hao Tseng, Chia-Wei Liu, Po-Yu Liu

**Affiliations:** 1 Division of Infectious Diseases, Taichung Veterans General Hospital, Taichung 40705, Taiwan; tedi3tedi3@vghtc.gov.tw (C.-H.T.); alice790305@vghtc.gov.tw (C.-W.L.); 2 Ph.D. Program in Translational Medicine, National Chung Hsing University, Taichung 40227, Taiwan; 3 Department of Post-Baccalaureate Medicine, College of Medicine, National Chung Hsing University, Taichung 40227, Taiwan; 4 Rong Hsing Research Center for Translational Medicine, National Chung Hsing University, Taichung 40227, Taiwan; 5 Genomic Center for Infectious Diseases, Taichung Veterans General Hospital, Taichung 40705, Taiwan

**Keywords:** antimicrobial-resistant bacteria, horizontal transmission, resistance genes

## Abstract

Animals have been identified as potential reservoirs and vectors of resistance genes, with studies showing that Gram-negative bacteria can acquire resistance through the horizontal transmission of resistance genes on plasmids. It is important to understand the distribution of antimicrobial-resistant bacteria and their drug-resistant genes in animals. Previous review articles mostly focused on a single bacterium or a single animal. Our objective is to compile all ESBL-producing bacteria isolated from various animals in recent years and provide a comprehensive viewpoint. Using a thorough PubMed literature search spanning from 1 January 2020 to 30 June 2022, studies exploring extended-spectrum beta-lactamase (ESBL) producing bacteria in animals were included. ESBL-producing bacteria are present in animals from various countries around the world. The most common sources of these bacteria were farm animals, and the most frequently isolated bacteria were *Escherichia coli* and *Klebsiella pneumoniae*. The most detected ESBL genes were *bla*_TEM_, *bla*_SHV_, and *bla*_CTX-M_. The presence of ESBL-producing bacteria in animals highlights the importance of the One Health approach to address the issue of antibiotic resistance. Further research is needed to better understand the epidemiology and mechanisms of the spread of ESBL-producing bacteria in animal populations and their potential impact on human and animal health.

## 1. Introduction

Antibiotics are important weapons for humans in fighting microbial infections and reducing overall mortality from infectious diseases. However, the increasing prevalence of antimicrobial-resistant bacteria (AMRs) in recent decades is a great challenge [1]. Studies suggest that animals are potential reservoirs and vectors of resistance genes [2]. Gram-negative bacteria (GNB), especially Enterobacterales strains, can acquire resistance through the plasmid-mediated horizontal transmission of resistance genes [3]. Increased use of antibiotics in livestock has been identified as a potential contributor to antimicrobial resistance in humans [4]. Therefore, it is also important to understand the distribution of antimicrobial-resistant bacteria and their drug-resistant genes in animals.

Bacteria that produce extended-spectrum β-lactamases (ESBLs) are considered one of the critical priority pathogens by the World Health Organization (WHO). ESBLs are a type of β-lactamase that can hydrolyze penicillins, first, second, and third-generation cephalosporins, and aztreonam but are unable to break down cephamycins or carbapenems [5]. The ESBL-encoding genes can be grouped into several families: *bla*_TEM_, *bla*_SHV_, *bla*_CTX-M_, etc. In the past, TEM and SHV-type ESBLs were the mainstream of ESBLs. However, CTX-M-type enzymes are much more commonly found in the ESBL type in recent research [5].

The widespread use of antibiotics is a contributing factor in the rise of antimicrobial resistance, particularly in the case of ESBL-producing bacteria. Previous studies have shown that the use of antibiotics within the past three months and monotherapy with specific drug classes (cephalosporins, tetracycline, macrolide, and cotrimoxazole) are associated with the prevalence of these bacteria [6,7]. The plasmids responsible for ESBL production often carry genes encoding resistance to other drug classes, such as aminoglycosides, trimethoprim, and fluoroquinolones [8]. This makes the treatment of infections caused by ESBL-producing bacteria more challenging, as the presence of these plasmids exacerbates the problem of antibiotic resistance and limits therapeutic options. In short, the overuse of antibiotics creates a favorable environment for the spread of plasmids responsible for ESBL production, which in turn contributes to the rise of antibiotic resistance.

Animals have been identified as potential reservoirs and vectors of resistance genes. The widespread use of antibiotics in animals has been linked to the increasing prevalence of antimicrobial-resistant bacteria in humans, highlighting the importance of understanding the distribution of antimicrobial-resistant bacteria and their drug-resistant genes in both humans and animals.

There are many articles on the analysis of ESBL-producing bacteria and their drug-resistance genes in particular animals. While some review articles have attempted to summarize these studies, most of them have focused on a specific type of bacteria (such as *Escherichia coli* or *Klebsiella pneumoniae*) or only reviewed one type of animal, lacking a comprehensive review. Our objective is to compile all ESBL-producing bacteria isolated from various animals in recent years, providing a comprehensive understanding of the distribution of ESBL-producing bacteria and genes in animals worldwide. This review attempts to underscore the role of animals in the rising incidence of ESBL-producing bacteria and the need for a coordinated effort to address this growing threat.

## 2. Results

The general findings of the reviewed articles are summarized in Table 1. Samples of ESBL-producing bacteria were mostly obtained from farms in Africa (Egypt, Kenya, Tunisia, Nigeria, and Algeria), Asia (Pakistan, India, Qatar, Iran, Malaysia, China, Saudi Arabia, Bangladesh, and Thailand), Europe (Finland, Portugal, Spain, Netherlands, Germany, France, and Switzerland), North American (USA), and South America (Brazil, Guadeloupe, and Peru). Other sampling locations included the airport, animal clinics, animal shelters, hunting grounds, petting zoos, slaughterhouses, research facilities, universities, and wild colonies. Most samples were obtained from rectal swabs and fresh feces of animals. However, other samples including raw milk, blood and visceral samples, cloacal swabs, uterine swabs, external surface and gut homogenates, urine, pus, and respiratory pathological specimens were also included. The most reported bacteria were Escherichia coli and Klebsiella pneumoniae. Other Enterobacterales were also in abundance while Pseudomonas aeruginosa was only found on the uterine swabs of farm cows, camels, and mares in one study from Saudi Arabia.

**Figure 1:**
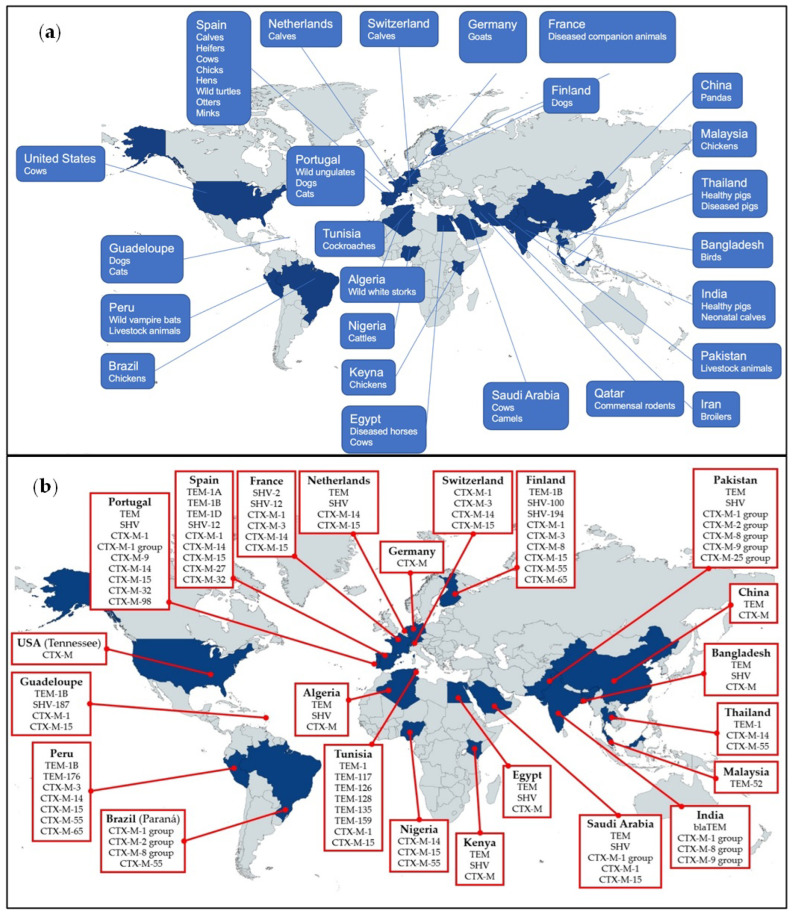
(**a**) Countries that reported the presence of ESBL-producing bacteria in animals. (**b**) Countries that reported the presence of ESBL-subtype distribution in animals.

Our review included 23 articles on domestic animals, 6 articles on wild animals, and 1 article on both. Other than farm animals, pets, zoo animals, vampire bats, and cockroaches were sampled. Four studies emphasized that the specimens were sourced from diseased animals, including diseased companion animals, diseased horses, diseased cows, camels, mares, and diseased pigs. The compilation of animals screened across different countries is presented in Figure 1a.

Table 2 summarizes the details of the ESBL genes. Most samples were grown using MacConkey agar. Fifteen articles included in our review utilized selective media supplemented with third-generation cephalosporins for initial ESBL screening. Most targeted bacteria were identified by Matrix-Assisted Laser Desorption Ionization–Time-of-Flight (MALDI-TOF) and polymerase chain reaction (PCR). The prevalence of ESBL in the samples varied widely from 0 to 100%. Double-disc synergy test was mostly used for identifying ESBL-producing bacteria. The most detected ESBL genes were *bla*_TEM_, *bla*_SHV_, and *bla*_CTX_-_M_. Subtype distribution around the world can be found in Figure 1b. Primers used in the reviewed articles are listed in Table 3. No standardized primer was used for each target gene. However, Woodford et al. (2006) was the most highly cited article for primers targeting specific groups of *bla*_CTX-M_.

## 3. Discussion

The results of the literature review provide a comprehensive comparison with previous studies on ESBL-producing bacteria in animals. While most previous studies have focused on limited geographic regions and animal populations, the current literature review offers a broader perspective on the highly diverse nature of ESBL-producing bacteria. This review provides insights into the distribution and occurrence of ESBL-producing bacteria in different regions and animal populations, helping to fill gaps in our understanding of this important issue.

Although previous studies have established the spread of ESBL-producing Enterobacterales in food-producing animals and companion pets around the world [67,68], this review highlights the presence of ESBL-producing bacteria in wild vampire bats, wild ungulates, and cockroaches. These findings suggest that the spread of ESBL-producing bacteria is not limited to domesticated animals, but can also occur in wild animal populations. The presence of ESBL-producing bacteria in wild animals can have significant implications for their health, as well as for the health of other animal populations and humans that may encounter them. It is important to consider the potential sources of ESBL-producing bacteria in wild animals, including exposure to contaminated food and water sources, contact with domesticated animals and their environment, or exposure to antibiotics in the environment [69,70]. The spread of ESBL-producing bacteria in wild animal populations can also have ecological consequences, such as altering the balance of microbial communities and affecting the health of the animals and their habitat. It is crucial to continue monitoring the presence of ESBL-producing bacteria in wild animal populations and to implement strategies to reduce their spread. The One Health approach, which recognizes the interconnections between human, animal, and environmental health, is crucial in addressing the issue of ESBL-producing bacteria in wild animals. In the past, WHO had launched an integrated global surveillance on ESBL-producing *E. coli*. with the same approach [71]. Similar programs covering a wider range of ESBL-producing bacteria may be considered.

Samples in this review were mostly obtained from Enterobacterales-rich areas such as the rectum and fresh feces. However, ESBL-producing bacteria were also found in raw milk and blood and visceral samples of animals in this review [15,17,20]. Unlike most other samples where ESBL-producing genes were found in Enterobacterales, one study reviewed showed the presence of *Pseudomonas aeruginosa* in uterine swabs of farm animals [28]. The potential for animals to act as reservoirs and vectors of resistance genes is therefore not limited to Enterobacterales found in food-producing animals and pets in contact with humans and antibiotics. One study in Kenya was conducted to collect cloacal swabs from the chickens and fecal samples from the farms. Out of the 544 cloacal isolates of Enterobacterales, 30 were found to contain ESBL genes. Among these, 14 isolates had *bla*_TEM_, 5 had *bla*_SHV_, and 11 had *bla*_CTX-M_. In contrast, among the 47 human isolates, 3 were found to contain ESBL genes, including 2 with *bla*_TEM_ and 1 with *bla*_CTX-M_ [25].

There are many kinds of ESBL-encoding genes, including *bla*_TEM_, *bla*_SHV_, *bla*_CTX_-_M_, *bla*_GES_, *bla*_VEB_, *bla*_IRT_, *bla*_CMT_, *bla*_BEL_, *bla*_TLA_, and *bla*_PER_ [5]. However, most studies reviewed that investigated bacteria from animals only screened for *bla*_TEM_, *bla*_SHV_, and *bla*_CTX-M_. Genes including *bla*_GES_, *bla*_VEB_, *bla*_IRT_, *bla*_CMT_, *bla*_BEL_, *bla*_TLA_, and *bla*_PER_ were rarely described and did not occur as frequently as the former three. This is possibly due to the genes being encoded on the chromosomes and not plasmids [8,72]. Furthermore, researchers used various primers for the detection and sequencing of the target genes. The results of this review draw attention to the need for standardized and comprehensive surveillance of ESBL-producing bacteria in animal populations. The limited screening for only a few types of ESBL-encoding genes, such as *bla*_TEM_, *bla*_SHV_, and *bla*_CTX-M_, may not fully capture the diversity and distribution of ESBL-producing bacteria in animal populations. Standardized surveillance covering a wider range of animals and regions will be necessary to better understand the spread of ESBL-producing bacteria and the potential impact on human and animal health. In addition, the use of different primers for the detection and sequencing of ESBL-encoding genes can lead to variability in the results and limit the comparability of studies [73]. Standardized protocols for the detection and sequencing of ESBL-encoding genes are necessary to ensure consistent and accurate results, which is the cornerstone of a better understanding of the spread of these bacteria and their potential impact on human and animal health.

In recent years, there has been a growing concern over the emergence of *bla*_ESBL_-harboring plasmids in animal isolates. These plasmids are capable of transmitting *bla*_ESBL_ genes among different bacterial species and even among different hosts, including animals and humans [74,75]. Studies have shown that *bla*_ESBL_-harboring plasmids can be found in various animal isolates, including those from bovine, camels, dogs, cats, goats, and poultry [10,14,76,77,78]. These plasmids can spread rapidly across and within bacterial populations, leading to the dissemination of antibiotic-resistance genes and the emergence of multidrug-resistant bacteria [79]. Overall, the occurrence of *bla*_ESBL_-harboring plasmids in animal isolates highlights the need for effective surveillance and control measures to limit the spread of antibiotic resistance in both animal and human populations.

PCR with oligonucleotide primers that are specific for a β-lactamase gene was the most common and simplest molecular method used to identify the presence of a β-lactamase enzyme belonging to a specific family. The chosen primers were designed to anneal to regions where no point mutations were known to occur [8,72]. However, in some cases, specific primers may have had special restrictions. For example, the *bla*_CTX-M_ primer Forward (5′-CGCTTTGCGATGTGCAG-3′) and Reverse (5′-ACCGCGATATCGTTGGT-3′) should not be used to detect CTX-M enzymes in *Klebsiella oxytoca* as it would result in amplifying chromosomal *bla*_oxy_ genes [80]. However, the direct comparison of the sensitivity and specificity of different primers in detecting specific ESBL genes is lacking. Therefore, exploring the impact of different primers on blaESBL epidemiology may be a potential research direction.

Though the presence of ESBL-producing bacteria could be seen around the world from this review, regions such as Australia, Canada, and Russia were not covered. This may be due to the fewer numbers of literature reviewed. The prevalence of the ESBL-producing bacteria ranged from 0–100% in this review. Results varied widely among different species and regions. Further research in different regions and animal populations is needed to gain a more comprehensive understanding of the distribution of ESBL-producing bacteria in animals. To limit the spread of ESBL-producing bacteria in animals, the development of new strategies, including the use of alternative treatments, improved animal husbandry practices, and increased public awareness are also vital.

Our review is subject to significant limitations. Specifically, our use of only one database (PubMed) and a limited set of keywords, as well as our exclusion of non-English literature. These may have resulted in some relevant publications being overlooked.

## 4. Materials and Methods

### 4.1. Literature Search Strategy

Using a thorough PubMed literature search from 1 January 2020 to 30 June 2022, studies that investigated bacteria from animals (whether wild or domestic) with details of ESBLs were included along with current contents and references from relevant articles. We combined the medical MeSH terminology with free-text terms to conduct a systematic literature search. These were the four keyword combinations we used to search: [(Animals) AND (extended-spectrum beta-lactamase)], [(Animals) AND ESBL], [“Animals” [Mesh] AND (extended-spectrum beta-lactamase)], and [“Animals” [Mesh] AND ESBL]. The bibliographic search was carried out by two researchers. The review protocol is provided as Supplementary Materials File S1.

### 4.2. Inclusion and Exclusion Criteria

The inclusion criteria include: (1) cultivation of bacteria from animal specimens, whether the animal is healthy or sick; (2) conducting ESBL testing on the bacteria, whether it is phenotype or genotype; and (3) the language of publication was English. The exclusion criteria were as follows: (1) specimens from humans or the surrounding environment of animals were excluded; (2) specimens from animals, humans, and the environment, with no clear distinction between them, were also excluded; and (3) specimens that may be contaminated by environmental or human factors were excluded, such as dairy products, supermarket-packaged raw meat, poultry litter, and pooled feces. However, raw milk and fresh feces are acceptable specimens, as we believe the probability of bacteria cultured from these two types of specimens being contaminated by environmental or human factors is low.

### 4.3. Study Selection

**Figure 2:**
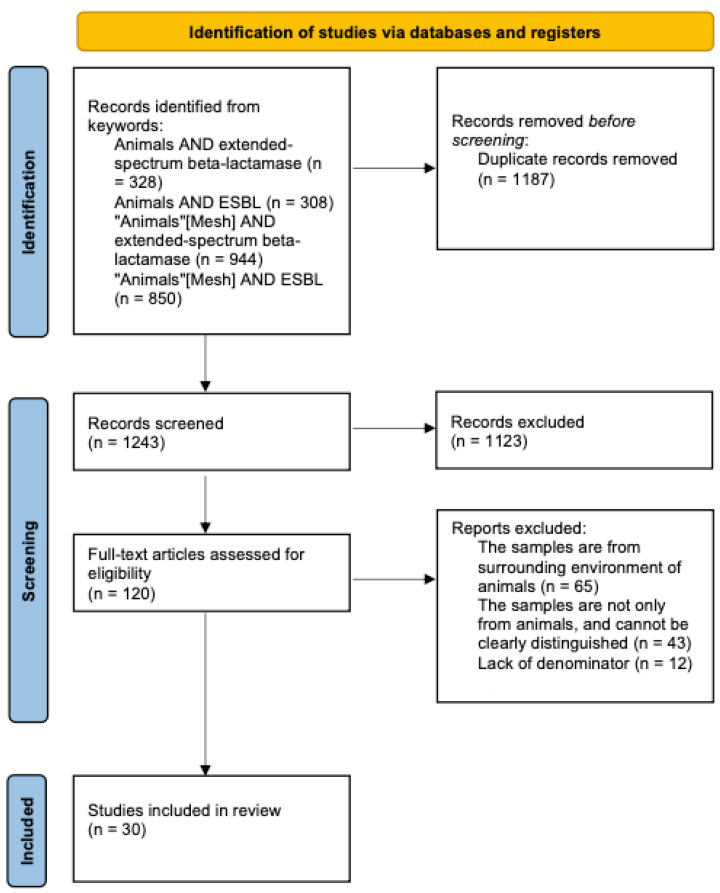
Flow diagram of the study selection process.

Our search uncovered 2430 bibliographic references to articles published between 1 January 2021 and 30 June 2022. Thereafter, 1187 duplicate records were removed. Finally, 1243 references remained for screening. The PRISMA 2020 flow diagram [81] for literature screening can be viewed in Figure 2. After the screening of the 1243 records, 1123 records did not match the type of articles we wanted to include. Hence, 120 pieces of full-text literature were assessed for eligibility. Sixty-five of them were excluded because the samples were from the surrounding environment of animals, not from animals themselves. Forty-three of them were excluded because the samples were not only from animals but also from humans or the environment, which could not be distinguished based on the content of the article. Twelve articles were excluded because no denominator (whether the number of animals, number of samples, or number of cultured bacteria) was provided. Finally, 30 documents were selected for further review and analysis.

### 4.4. Data Extraction

We extracted data from all selected literature using a standardized table. The data were grouped as follows: author, date of publication, countries, sampling date and location, sample type, animal species, targeted bacteria, selective media, methods for target identification, the number of denominators, the number of ESBL target, methods for detecting ESBL, methods for detecting ESBL genes, and the number of particular ESBL genes. The collected data were entered into standardized data extraction sheets using Microsoft Excel 2019 (Microsoft Corp, Seattle, WA, USA) for data extraction.

## 5. Conclusion

The results of this systematic literature review show that ESBL-producing bacteria are present in animals from various countries around the world. We focused on articles where samples were obtained from animals, excluding data from the environment or humans. The most common sources of these bacteria were farm animals, and the most frequently isolated bacteria were *E. coli* and *K. pneumoniae*. The prevalence of ESBL in the samples varied widely and the most commonly detected ESBL genes were *bla*_TEM_, *bla*_SHV_, and *bla*_CTX-M_.

The presence of ESBL-producing bacteria in animals highlights the importance of the One Health approach to address the issue of antibiotic resistance. Further research is needed to better understand the epidemiology and mechanisms of the spread of ESBL-producing bacteria in animal populations and their potential impact on human and animal health.

## Figures and Tables

**Table 1:** Studies of ESBL-producing bacteria in animals.

Article	Country	Location	Sample	Animals	Date of Isolation	Bacteria
**Venla Johansson et al., 2022** [9]	Finland	airport, animal clinics, animal shelters	rectal swabs or fresh feces	dogs	2017–2018	*Escherichia coli* and *Klebsiella pneumoniae*
**Muhammad Shafiq et al., 2022** [10]	Pakistan	farms	rectal swabs or fresh droppings	buffaloes, cattle, sheep, goats, and broilers	no details	*Escherichia coli*
**Rita Tinoco Torres et al., 2022** [11]	Portugal	hunting grounds	rectal swabs	Wild ungulates	October 2018–February 2020	Enterobacterales
**Maitane Tello et al., 2022** [12]	Spain	dairy cattle farms	rectal swabs	calves, heifers, and cows	February 2019–October 2020	*Escherichia coli*
**Tilaye Shibbiru Mengistu et al., 2022** [13]	Spain	a highly populated and intensive farming region	cloacal/rectal swabs	turtles, minks, and otters	January 2018–July 2021	Enterobacterales, and some other Gram-negative bacteria
**Irene Aldea et al., 2022** [14]	Spain	a commercial laying hen farm	fresh meconium droppings, feces	chicks and hens	March 2016–October 2018	*Escherichia coli*
**Rasha Elkenany et al., 2022** [15]	Egypt	dairy farms	raw cow milk	cows	2018	*Shigella* species
**Teresita d.J. Bello Gonzalez et al., 2022** [16]	Netherlands	dairy farms	rectal swabs	calves	March 2019–May 2020	*Escherichia coli* and *Klebsiella pneumoniae*
**Benti D Gelalcha et al., 2022** [17]	USA	dairy farms	bulk tank milk	cows	no details	*Escherichia coli*
**Jannis Göttling et al., 2022** [18]	Germany	petting zoo	rectal swabs	healthy goats	August 2016–June 2017	Enterobacterales
**Nagappa Karabasanavar et al., 2022** [19]	India	pig farms	rectal swabs	healthy pigs	April 2019–April 2020	*Salmonella* species
**Md Mazharul Islam et al., 2021** [20]	Qatar	livestock farms, agricultural farms	blood and visceral samples	commensal rodents	August 2019–February 2020	Gram-negative bacilli
**Damini P. Khawaskar et al., 2021** [21]	India	dairy farms	rectal swabs	neonatal calves	no details	*Escherichia coli*
**Mehri Haeili et al., 2021** [22]	Iran	a chicken slaughterhouse	cloacal swabs	slaughtered broilers	no details	*Escherichia coli* and *Klebsiella pneumoniae*
**Erkihun Aklilu et al., 2022** [23]	Malaysia	farms	cloacal swabs	broiler chickens	no details	*Escherichia coli*
**Maísa Fabiana Menck-Costa et al., 2022** [24]	Brazil	broiler farms	cloacal swabs	broiler chickens	March 2019–July 2020	*Escherichia coli*
**James G Ndukui et al., 2021** [25]	Kenya	poultry production centers	cloacal swabs	chickens	November 2020–February 2021	Enterobacterales
**Xiaoyan Su et al., 2022** [26]	China	Chengdu Research Base of Giant Panda Breeding	fresh feces	captive giant pandas	2018–2019	*Klebsiella pneumoniae*
**Gaëlle Gruel et al., 2022** [27]	Guadeloupe (French West Indies)	animal shelters and veterinary clinics	rectal swabs	dogs and cats	June 2019–September 2019	Enterobacterales
**Samy F. Mahmoud et al., 2022** [28]	Saudi Arabia	farms	uterine swabs	diseased cow, camel, and mare	May 2020–February 2021	*Pseudomonas aeruginosa*
**Md Saiful Islam et al., 2021** [29]	Bangladesh	no details	fresh feces	migratory birds	November 2019–November 2020	*Escherichia coli*
**Sarrah Landolsi et al., 2022** [30]	Tunisia	a collective catering, houses, and a hospital	external surface and gut homogenates	cockroaches	July 2017–June 2018	Enterobacterales
**Raquel Garcia-Fierro et al., 2022** [31]	France	no details	Urine, pus, and respiratory pathological specimens	diseased dogs, cats, horses, cattle, and birds	2010–2018	*Klebsiella pneumoniae*
**Adriana Belas et al., 2022** [32]	Portugal	faculty of veterinary medicine	urine	dogs and cats	1999–2015	*Escherichia coli*
**Mabel Kamweli Aworh et al., 2022** [33]	Nigeria	abattoirs	Cecal contents from the cecum	slaughtered beef cattle	May 2020–December 2020	*Escherichia coli*
**Magdalena Nüesch-Inderbinen et al., 2022** [34]	Switzerland	organic and conventional dairy farms	fresh feces	calves	September 2020	Enterobacterales
**Ahmed Samir et al., 2022** [35]	Egypt	equine farms	rectal swabs and nasal swabs	diseased adult horses	August 2020–March 2021	*Klebsiella pneumoniae*
**Lotfi Loucif et al., 2021** [36]	Algeria	nests and a colony	fresh feces	white stork	May 2019	Enterobacterales
**Julio A Benavides et al., 2021** [37]	Peru	colonies	rectal swabs	vampire bats	2015 (October), 2017 (March to May), and 2018 (February and March)	*Escherichia coli*
		farms located nearby vampire bat colonies	fresh feces	cows, pigs, goats, sheep, and donkeys	2015	*Escherichia coli*
**Suthathip Trongjit et al., 2022** [38]	Thailand	farms	rectal swabs	pigs	2007–2018	*Escherichia coli*

**Table 2:** Characteristics of ESBL genes in animals in the review.

Article	Selective Media	Target Identification	Total Number	ESBL Number	%	ESBL Test	ESBL Genes Test	ESBL Genes and Number
**Venla Johansson et al., 2022 [9]**	MacConkey agar with cefotaxime (1 mg/L)	MALDI-TOF	60	47	78.3%	double-disc synergy test	WGS	*bla*_TEM_ (25), *bla*_SHV_ (2), *bla*_CTX-M-1_ (7), *bla*_CTX-M-3_ (1), *bla*_CTX-M-15_ (36), *bla*_CTX-M-55_ (2), *bla*_CTX-M-8_ (1), *bla*_CTX-M-65_ (1)
**Muhammad Shafiq et al., 2022 [10]**	MacConkey agar	PCR (uidA gene)	153	75	49.0%	double-disc synergy test	PCR	*bla*_TEM_ (37), *bla*_SHV_ (32), *bla*_CTX-M-1_ group (35), *bla*_CTX-M-2_ group (5), *bla*_CTX-M-8_ group (1), *bla*_CTX-M-9_ group (32), *bla*_CTX-M-25_ group (3)
**Rita Tinoco Torres et al., 2022 [11]**	MacConkey agar with antibiotic ^1^	biochemical reaction (API20E galleries)	151	4	2.6%	Characteristic phenotypic synergism with ESBL genes	PCR	*bla*_TEM_ (60), *bla*_SHV_ (3), *bla*_CTX-M_ (4) [_CTX-M-14_ (2), _CTX_-_M-15_ (1), _CTX-M-98_ (1)]
**Maitane Tello et al., 2022 [12]**	MacConkey agar with cefotaxime (1 mg/L)	PCR (uidA gene)	41	39	95.1%	ESBL genes	WGS	*bla*_TEM_ (17), *bla*_SHV_ (1), *bla*_CTX-M-1_ (9), *bla*_CTX-M-14_ (12), *bla*_CTX-M-15_ (9), *bla*_CTX-M-27_ (3), *bla*_CTX-M-32_ (5)
**Tilaye Shibbiru Mengistu et al., 2022 [13]**	MacConkey agar with ceftriaxone (1 mg/L)	API^®^ biochemical test strips or automated system (VITEK 2)	131	4	3.1%	ESBL genes	PCR	*bla*_TEM_ (0), *bla*_CTX-M_ (4) [_CTX-M-15_ (4)]
**Irene Aldea et al., 2022 [14]**	MacConkey agar with cefotaxime (1 mg/L)	PCR, API 20-E kit, or whole genome sequencing	47	29	61.7%	ESBL genes ^8^	WGS	*bla*_TEM_ (19), *bla*_SHV_ (9), *bla*_CTX-M-1_ (19), *bla*_CTX-M-14_ (1)
**Rasha Elkenany et al., 2022 [15]**	Salmonella-Shigella agar, MacConkey agar, and xylose-lysine-deoxycholate agar	Biochemical reaction ^4^	16	4	25.0%	double-disc synergy test	PCR	*bla*_TEM_ (16), *bla*_SHV_ (0), *bla*_CTX-M_ (4)
**Teresita d.J. Bello Gonzalez et al., 2022 [16]**	MacConkey agar with cefotaxime (1 mg/L)	MALDI-TOF	254	254	100.0%	ESBL genes	PCR	*bla*_TEM_ (254), *bla*_SHV_ (174), *bla*_CTX-M-14_ (174), *bla*_CTX-M-15_ (80)
**Benti D Gelalcha et al., 2022 [17]**	CHROMagar™ *E. coli* agar	PCR (uidA gene)	14	4	28.6%	ESBL genes	PCR	*bla*_TEM_ (0), *bla*_SHV_ (0), *bla*_CTX-M_ (4)
**Jannis Göttling et al., 2022 [18]**	Oxoid Brilliance ESBL agar	automated system (VITEK 2)	300	1	0.3%	Commercial disc test system (D68C ESBL/AmpC ID, MAST group Diagnostics)	PCR	*bla*_TEM_ (0), *bla*_SHV_ (0), *bla*_CTX-M-1_ (1), *bla*_CTX-M-2_ (0), *bla*_CTX-M-9_ (0)
**Nagappa Karabasanavar et al., 2022 [19]**	Xylose-lysine-deoxycholate agar, Brilliant green agar, Bismuth sulfite agar, Hektoen Enteric agar	Biochemical reaction ^5^	22	12	54.5%	ESBL genes	PCR	*bla*_TEM_ (12), *bla*_SHV_ (0), *bla*_CTX-M-1_ (0), *bla*_CTX-M-2_ (0), *bla*_CTX-M-9_ (0)
**Md Mazharul Islam et al., 2021 [20]**	MacConkey agar, Hektoen enteric agar, Eosin methylene blue agar	automated system (VITEK 2)	68	9	13.2%	VITEK 2 AST-GN cards	no test	no test
**Damini P. Khawaskar et al., 2021 [21]**	MacConkey agar and Eosin methylene blue agar	biochemical reaction (IMViC Test)	280	120	42.9%	combination disk method	PCR	*bla*_TEM_ (10), *bla*_SHV_ (0), *bla*_CTX-M-1_ group (34), *bla*_CTX-M-2_ group (0), *bla*_CTX-M-8_ group (2), *bla*_CTX-M-9_ group (1), *bla*_CTX-M-25_ group (0)
**Mehri Haeili et al., 2021 [22]**	no details	no details	21	0	0.0%	combination disk method	no test	no test
**Erkihun Aklilu et al., 2022 [23]**	MacConkey and Eosine Methylene Blue agars	PCR (*E. coli* specific gene)	49	12	24.5%	ESBL genes	PCR	*bla*_TEM_ (12), *bla*_CTX-M_ (0)
**Maísa Fabiana Menck-Costa et al., 2022 [24]**	MacConkey agar with/without antibiotics ^2^	biochemical reaction ^6^	360	198	55.0%	double-disc synergy test	PCR	*bla*_CTX-M-1_ group (153), *bla*_CTX-M-2_ group (61), *bla*_CTX-M-8_ group (5), *bla*_CTX-M-9_ group (0), *bla*_CTX-M-25_ group (0)
**James G Ndukui et al., 2021 [25]**	no details	biochemical reaction	544	30	5.5%	phenotypic resistance profiles and then ESBL genes	PCR	*bla*_TEM_ (14), *bla*_SHV_ (5), *bla*_CTX-M_ (11)
**Xiaoyan Su et al., 2022 [26]**	no details	16 s rDNA and biochemical reaction	211	3	1.4%	double-disc synergy test	PCR	*bla*_TEM_ (2), *bla*_SHV_ (0), *bla*_CTX-M_ (3), *bla*_GES_ (0), *bla*_PER_ (0), *bla*_VEB_ (0)
**Gaëlle Gruel et al., 2022 [27]**	CHROMagar™ CCA with ceftriaxone (4 mg/L)	API 20-E kit	185	14	7.6%	double-disk synergy test	WGS	*bla*_TEM_ (1), *bla*_SHV_ (1), *bla*_CTX-M-1_ (11), *bla*_CTX-M-15_ (3)
**Samy F. Mahmoud et al., 2022 [28]**	Pseudomonas cetrimide agar	automated system (VITEK 2)	44	20	45.5%	double-disk synergy test	PCR	*bla*_TEM_ (18), *bla*_SHV_ (8), *bla*_CTX-M_ (11)
**Md Saiful Islam et al., 2021 [29]**	Eosin methylene blue agar	biochemical reaction ^7^	55	21	38.2%	double-disk synergy test	PCR	*bla*_TEM_ (20), *bla*_SHV_ (9), *bla*_CTX-M_ (18)
**Sarrah Landolsi et al., 2022 [30]**	MacConkey agar with cefotaxime (1 mg/L)	MALDI-TOF	144	22	15.3%	double-disk synergy test	PCR	*bla*_TEM_ (9), *bla*_SHV_ (0), *bla*_CTX-M_ (15) [*bla*_CTX-M-1_ (7), *bla*_CTX-M-15_ (8)]
**Raquel Garcia-Fierro et al., 2022 [31]**	no details, but cefoxitin- and/or ceftiofur-resistant	MALDI-TOF	105	52	49.5%	ESBL genes	WGS	*bla*_SHV_ (2), *bla*_CTX-M-1_ (3), *bla*_CTX-M-3_ (1), *bla*_CTX-M-14_ (4), *bla*_CTX-M-15_ (42)
**Adriana Belas et al., 2022 [32]**	no details, but Third-generation cephalosporin-resistant	PCR (gadA gene)	35	14	40.0%	ESBL genes	PCR	*bla*_CTX-M-1_ (2), *bla*_CTX-M-1-like_ (2), *bla*_CTX-M-9_ (1), *bla*_CTX-M-15_ (7), *bla*_CTX-M-32_ (3)
**Mabel Kamweli Aworh et al., 2022 [33]**	MacConkey agar with cefotaxime (1 mg/L)	biochemical reaction (commercially Microbact GNB 24E kit)	272	44	16.2%	combination disk method	WGS	*bla*_CTX-M-14_ (1), *bla*_CTX-M-15_ (41), *bla*_CTX-M-55_ (1)
**Magdalena Nüesch-Inderbinen et al., 2022 [34]**	Rapid’ *E. coli* two agar plates	MALDI-TOF	196	21	10.7%	Brilliance ESBL agar plates	PCR	*bla*_TEM_ (0), *bla*_SHV_ (0), *bla*_CTX-M-1_ (7), *bla*_CTX-M-3_ (4), *bla*_CTX-M-14_ (2), *bla*_CTX-M-15_ (8)
**Ahmed Samir et al., 2022 [35]**	MacConkey agar with cefotaxime (2 mg/L)	PCR (Klebsiella gyrA gene, ITS gene)	100	13	13.0%	double-disc synergy test	PCR	*bla*_TEM_ (13), *bla*_SHV_ (13), *bla*_CTX-M_ (12)
**Lotfi Loucif et al., 2021 [36]**	MacConkey agar with antibiotics ^3^	MALDI-TOF	42	8	19.0%	double-disc synergy test	PCR	*bla*_TEM_ (20), *bla*_SHV_ (4), *bla*_CTX-M_ (19)
**Julio A Benavides et al., 2021 [37]**	ChromID ESBL agar	MALDI-TOF	388	20	5.2%	ChromID ESBL agar	WGS	*bla*_TEM_ (17), *bla*_CTX-M-3_ (2), *bla*_CTX-M-14_ (0), *bla*_CTX-M-15_ (7), *bla*_CTX-M-55_ (8), *bla*_CTX-M-65_ (1)
			134	65	48.5%	ChromID ESBL agar	WGS	*bla*_TEM_ (14), *bla*_CTX-M-3_ (1), *bla*_CTX-M-14_ (3), *bla*_CTX-M-15_ (2), *bla*_CTX-M-55_ (7), *bla*_CTX-M-65_ (3)
**Suthathip Trongjit et al., 2022 [38]**	no details	no details	454	112	24.7%	combination disk method	PCR	*bla*_TEM_ (81), *bla*_SHV_ (0), *bla*_CTX-M-14_ (61), *bla*_CTX-M-55_ (48)

MALDI-TOF: Matrix-Assisted Laser Desorption Ionization–Time-of-Flight, PCR: polymerase chain reaction, WGS: whole genome sequencing. ^1^ Including ampicillin (100 μg/mL), cefotaxime (1 μg/mL), meropenem (0.5 μg/mL), ciprofloxacin (1 μg/mL), or tetracycline (100 μg/mL). ^2^ Ciprofloxacin, cefotaxime, and ciprofloxacin + cefotaxime, at a final concentration of 8 mg/mL. ^3^ 2 μg/mL cefotaxime, 2 μg/mL ertapenem, 9 μg/mL imipenem, and 3 μg/mL colistin, respectively. ^4^ Triple sugar iron agar, lysine iron agar, methyl red, Voges–Proskauer broth, the indole test, urea agar, Simmon’s citrate agar, and a motility test. ^5^ Methyl red, Voges–Proskauer, indole, Simmon’s citrate, urease, triple sugar iron agar, lysine decarboxylase, phenol red dulcitol, KCN, and malonate. ^6^ Triple sugar iron agar, indole production, Simmon’s citrate, urease production, lysine decarboxylation, and sorbitol and cellobiose fermentation tests. ^7^ Catalase test, coagulase test, sugar fermentation tests, methyl red test, Voges–Proskauer test, and indole test. ^8^ This article does not classify *bla*_TEM_ as an ESBL gene.

**Table 3:** Primers used for detecting ESBL-encoding genes in the review.

Target	Forward Primer (5′-3′)	Reverse Primer (5′-3′)	Articles	Reference
***bla*_TEM_**	CATTTCCGTGTCGCCCTTATTC	CGTTCATCCATAGTTGCCTGAC	[11,19,26]	Dallenne et al., 2010 [39]
	CATTTCCGTGTCGCCCTTATTC	TCCATAGTTGCCTGACTCCC	[29]	Randall et al., 2004 [40]
	CATTTCCGTGTCGCCCTTATTC	CCAATGCTTAATCAGTGAGGC	[17]	Strauss et al., 2015 [41]
	ATGAGTATTCAACATTTCCG	CCAATGCTTAATCAGTGAGGC	[25]	Gootz et al., 2009 [42]
	ATGAGTATTCAACATTTCCG	CTGACAGTTACCAATGCTTA	[21,30]	Bhattacharjee et al., 2007 [43], Christophy et al., 2017 [44]
	ATGAGTATTCAACATTTCCG	TTAATCAGTGAGGCACCTAT	[18]	Grobner et al., 2009 [45]
	TTCTGCTATGTGGTGCGGTA	GTCCTCCGATCGTTGTCAGA	[36]	Ly et al., 2019 [46]
	GCATCTTACGGATGGCATGA	GTCCTCCGATCGTTGTCAGA	[28]	Hosu et al., 2021 [47]
	TCGGGGAAATGTGCGCG	TGCTTAATCAGTGAGGCACC	[34]	Pitout et al., 1998 [48]
	CGCCGCATACACTATTCTCAGAATGA	ACGCTCACCGGCTCCAGATTTAT	[35]	Fang et al., 2008 [49]
	GCGGAACCCCTATTTG	TCTAAAGTATATATGAGTAAACTTGGTCTGAC	[13,38]	Darwich et al., 2019 [2], Hasman et al., 2005 [50]
	ATAAAATTCTTGAAGACGAAA	GACAGTTACCAATGCTTAATC	[10,23]	Ali et al., 2017 [51], Weill et al., 2004 [52]
	ATCAGCAATAAACCAGC	CCCCGAAGAACGTTTTC	[15]	Colom et al., 2003 [53]
***bla*_SHV_**	CACTCAAGGATGTATTGTG	TTAGCGTTGCCAGTGCTCG	[34]	Pitout et al., 1998 [48]
	TTCGCCTGTGTATTATCTCCCTG	TTAGCGTTGCCAGTGYTCG	[38]	Hasman et al., 2005 [50]
	GGGTTATTCTTATTTGTCGC	TTAGCGTTGCCAGTGCTC	[10]	Ali et al., 2017 [51]
	CTTTATCGGCCCTCACTCAA	AGGTGCTCATCATGGGAAAG	[35]	Fang et al., 2008 [49]
	TCCCATGATGAGCACCTTTAAA	TCCTGCTGGCGATAGTGGAT	[28,36]	Hosu et al., 2021 [47], Ly et al., 2019 [46]
	AGCCGCTTGAGCAAATTAAAC	ATCCCGCAGATAAATCACCAC	[11,19,26]	Dallenne et al., 2010 [39]
	AGGATTGACTGCCTTTTTG	ATTTGCTGATTTCGCTCG	[15]	Colom et al., 2003 [53]
	GCCGGGTTATTCTTATTTGTCGC	ATGCCGCCGCCAGTCA	[17]	Rayamajhi et al., 2008 [54]
	GCAAAACGCCGGGTTATTC	GGTTAGCGTTGCCAGTGCT	[18]	Grobner et al., 2009 [45]
	CCTTTAAAGTAGTGCTCTGC	TTCGCTGACCGGCGAGTAGT	[21]	Lob et al., 2015 [55]
	GGTTATGCGTTATATTCGCC	TTAGCGTTGCCAGTGCTC	[30]	Christophy et al., 2017 [44]
	ATGCGTTATWTTCGCCTGTGT	TTAGCGTTGGCAGTGCTCG	[25]	El-Shazly et al., 2015 [56]
	TCGCCTGTGTATTATCTCCC	CGCAGATAAATCACCACAATG	[29]	Van et al., 2008 [57]
***bla*_CTX-M_**	ATGTGCAGYACCAGTAARGTKATGGC	TGGGTRAARTARGTSACCAGAAYCAGCGG	[13,15,25,26,29,35,38]	Darwich et al., 2019 [2], Archambault et al., 2006 [58], Ahmed et al., 2013 [59], Su et al., 2022 [26], Gundran et al., 2019 [60], Fang et al., 2008 [49], Hasman et al., 2005 [50]
	ATGTGCAGYACCAGTAARGT	TGGGTRAARTARGTSACCAGA	[30]	Christophy et al., 2017 [44]
	CGATGTGCAGTACCAGTAA	TTAGTGACCAGAATCAGCGG	[38]	Batchelor et al., 2005 [61]
	TTTGCGATGTGCAGTACCAGTAA	CGATATCGTTGGTGGTGCCATA	[17,32]	Edelstein et al., 2003 [62]
	CGCTTTGCGATGTGCAG	ACCGCGATATCGTTGGT	[10,18]	Ali et al., 2017 [51], Grobner et al., 2009 [45]
	CCCATGGTTAAAAAACACTGC	CAGCGCTTTTGCCGTCTAAG	[23]	Horton et al., 2011 [63]
	ATGAGYACCAGTAARGTKATGGC	ATCACKCGGRTCGCCIGGRAT	[28]	Hosu et al., 2021 [47]
***bla*_CTX-M_ group 1**	AAAAATCACTGCGCCAGTTC	AGCTTATTCATCGCCACGTT	[11,21,24,32,34]	Woodford et al., 2006 [64]
	TTAGGAARTGTGCCGCTGYA	CGATATCGTTGGTGGTRCCAT	[19,38]	Dallenne et al., 2010 [39]
	GTTCGTCTCTTCCAGAATAAGG	CAGCACTTTTGCCGTCTAAG	[18]	Pfeifer et al., 2009 [65]
***bla*_CTX-M_ group 2**	CGACGCTACCCCTGCTATT	CCAGCGTCAGATTTTTCAGG	[11,21,24,32,34]	Woodford et al., 2006 [64]
	CGTTAACGGCACGATGAC	CGATATCGTTGGTGGTRCCAT	[19,38]	Dallenne et al., 2010 [39]
***bla*_CTX-M_ group 9**	CAAAGAGAGTGCAACGGATG	ATTGGAAAGCGTTCATCACC	[11,21,24,32,34]	Woodford et al., 2006 [64]
	TCAAGCCTGCCGATCTGGT	TGATTCTCGCCGCTGAAG	[19,38]	Dallenne et al., 2010 [39]
	ACACGGATTGACCGTATTGG	TGATTCTCGCCGCTGAAG	[18]	Wetzker et al., 2019 [66]
	GCAGTACAGCGACAATACCG	TATCATTGGTGGTGCCGTAG	[18]	Grobner et al., 2009 [45]
***bla*_CTX-M_ group 8**	TCGCGTTAAGCGGATGATGC	AACCCACGATGTGGGTAGC	[11,21,24,32,34]	Woodford et al., 2006 [64]
	AACRCRCAGACGCTCTAC	TCGAGCCGGAASGTGTYAT	[38]	Dallenne et al., 2010 [39]
***bla*_CTX-M_ group 25**	GCACGATGACATTCGGG	AACCCACGATGTGGGTAGC	[11,21,24,32,34]	Woodford et al., 2006 [64]
	AACRCRCAGACGCTCTAC	TCGAGCCGGAASGTGTYAT	[38]	Dallenne et al., 2010 [39]
***bla*_GES_**	AGTCGGCTAGACCGGAAAG	TTTGTCCGTGCTCAGGAT	[26]	Su et al., 2022 [26]
***bla*_PER_**	GCTCCGATAATGAAAGCG	TTCGGCTTGACTCGGCTGA	[26]	Su et al., 2022 [26]
***bla*_VEB_**	CATTTCCCGATGCAAAGCGT	CGAAGTTTCTTTGGACTCTG	[26]	Su et al., 2022 [26]

## Data Availability

Not applicable.

## Supplementary Materials

The following supporting information can be downloaded at: https://www.mdpi.com/article/10.3390/antibiotics12040661/s1, File S1: Review Protocol. Reference [82] is cited in the supplementary materials.
