# Supplementary material for: Extended-Spectrum β-Lactamases (ESBL) Producing Bacteria in Animals
Source: Antibiotics (Basel). 2023 Mar 28;12(4):661. doi: 10.3390/antibiotics12040661 (PMC10135299; doi:10.3390/antibiotics12040661)
Supplement: Supplementary file 1 [file antibiotics-12-00661-s001.zip › antibiotics-2275129-supplementary.pdf]

Supplementary Materials

File S1: Review Protocol

Project Information

**Review Title**	Extended-spectrum β-lactamases (ESBL) producing bacteria in animals
**Project Lead**	Po-Yu Liu
**Team Members**	Chien-Hao Tseng<br>Chia-Wei Liu
**Date**	2022/05 – 2023/02
**Institution(s)**	Taichung Veterans General Hospital

Background

Describe the population and problem or phenomenon of interest and contextualize it.

Animals have been identified as potential reservoirs and vectors of resistance genes, with studies showing that gram-negative bacteria can acquire resistance through the horizontal transmission of resistance genes on plasmids. It is important to understand the distribution of antimicrobial-resistant bacteria and their drug-resistant genes in both humans and animals.

Objective

Describe the justification for this review and why it/the information it collects is important.

There have been many studies reporting the presence of bla_{ESBL} in animals. While some review articles have attempted to summarize these studies, most of them have focused on a specific type of bacteria (such as Escherichia coli or Klebsiella pneumoniae) or only reviewed one type of animal, lacking a comprehensive review. Our objective is to compile all ESBL bacteria isolated from various animals in recent years, providing us with a comprehensive understanding of the distribution of ESBL bacteria and genes in animals worldwide.

Search Strategy

**Databases**<br>*List the bibliographic databases to be searched.*
Pubmed
**Hand Searching**<br>*List journals or websites that will be hand searched for relevant articles.*
Nil
**Experts or Stakeholders**

Review Protocol Template by Sarah Visintini is licensed under a Creative Commons Attribution-NonCommercial-ShareAlike 4.0 International License.

If experts are being contacted for additional grey literature or research, list them.
Nil
**Reference Searches**<br>*If forward or backward citations will be performed, detail them here.*
For the thirty articles we have collected that meet the inclusion criteria, we performed a second-level backward reference search.

Eligibility Criteria

Operationalize your PICO (or other framework) concepts by explicitly stating what would and would not meet inclusion.

PICO	Inclusion Criteria	Exclusion Criteria
Population	ESBL-producing bacteria	
Intervention	cultured from specimens of animal origin	Environment<br>human
Comparison	nil	
Outcomes	nil	

Data Extraction

Provide a description of methods used to collect data from included studies (e.g. categories of data you intend to collect, how many people will conduct extraction, etc.).

We extracted data from all selected literature using a standardized table. The data were grouped as follows: author, date of publication, countries, sampling date and lo-cation, sample type, animal species, targeted bacteria, selective media, methods for target identification, the number of denominators, the number of ESBL target, methods for detecting ESBL, methods for detecting ESBL genes and the number of particular ESBL genes. The collected data were entered into standardized data extraction sheets using Microsoft Excel 2019 (Microsoft Corp, Seattle, WA) for data extraction.

Review Protocol Template by Sarah Visintini is licensed under a Creative Commons Attribution-NonCommercial-ShareAlike 4.0 International License.

Study Quality Assessment

If applicable, describe the tool(s) you will use to assess risk of bias.

The quality of each study will be assessed using the Newcastle-Ottawa Scale.

Data Synthesis

Describe how you will analyze and summarize the included study results.

The extracted data will be synthesized using statistical methods such as meta-analysis, or if not feasible, may change to narrative synthesis. The heterogeneity between the studies will be assessed.

Project Tools

List the tools and software programs you plan to use in the course of the review.

PubMed: search articles<br>EndNote: create citations and reference lists<br>Excel: data extraction and analysis<br>PRISMA: provide a framework for our review

Project Timetable

Fill out the following Gantt chart according to your estimated project timelines.

	2022/5	2022/6	2022/7	2022/8	2022/9	2022/10	2022/11	2022/12	2023/1	2023/2
**Preparation**	■	■								
**Conduct searches**		■	■	■						
**Pilot test eligibility criteria**		■	■							
**Ti/Ab + Full Text Selection**		■	■	■	■	■				
**Pilot test data collection**				■	■					
**Data collection**					■	■	■			

Review Protocol Template by Sarah Visintini is licensed under a Creative Commons Attribution-NonCommercial-ShareAlike 4.0 International License.

**Pilot risk of bias assessments**										
**Conduct assessments**										
**Conduct synthesis & interpret results**										
**Write manuscript**										

Research Team Member Roles

Describe the different tasks on the review and who will be responsible for what.

Task	Description	Team Member Responsible
Conduct searches	searching the selected databases using the developed search terms and strategies	Po-Yu Liu<br>Chien-Hao Tseng
Pilot test eligibility criteria	testing the eligibility criteria on a small sample of studies to ensure that they are appropriate for the review	Po-Yu Liu<br>Chien-Hao Tseng
Full Text Selection	screening the titles and abstracts of studies retrieved from the search to identify potentially relevant studies, and then reviewing the full text of these studies to determine their eligibility for inclusion in the review	Po-Yu Liu<br>Chien-Hao Tseng<br>Chia-Wei Liu
Pilot test data collection	piloting the data extraction form on a small sample of studies to ensure that it captures all the necessary data	Po-Yu Liu<br>Chien-Hao Tseng
Data collection	extracting data from the included studies using the developed data extraction form	Chien-Hao Tseng<br>Chia-Wei Liu
Pilot risk of bias assessments	piloting the risk of bias assessment tool on a small sample of studies to ensure that it accurately assesses the quality of the included studies.	Po-Yu Liu<br>Chien-Hao Tseng
Conduct assessments	using the developed risk of bias assessment tool to assess the quality of the included studies	Chien-Hao Tseng<br>Chia-Wei Liu
Conduct synthesis & interpret results	synthesizing the data from the included studies and interpreting the results	Po-Yu Liu<br>Chien-Hao Tseng<br>Chia-Wei Liu
Write manuscript - Original Draft	writing the final manuscript for the review – original draft	Chien-Hao Tseng

Review Protocol Template by Sarah Visintini is licensed under a Creative Commons Attribution-NonCommercial-ShareAlike 4.0 International License.

Write manuscript - Review & Editing	writing the final manuscript for the review – review and editing	Po-Yu Liu

References

This Review Protocol was originally created by Sarah Visintini, Maritime SPOR SUPPORT Unit and was available from the following resources:

University of Alabama, University Libraries. A Research Guide for Systematic Literature Reviews. Available online: https://guides.lib.ua.edu/c.php?g=1135040&p=8286355 (accessed on 24 May 2022)

Additionally, this Systematic Review Protocol Template was adapted by Chien-Hao Tseng to suit the specific needs of our Review.

Review Protocol Template by Sarah Visintini is licensed under a Creative Commons Attribution-NonCommercial-ShareAlike 4.0 International License.
